# Effect of garlic powder supplementation on rumen microbiota and histology, and blood metabolites in Barki lambs

**DOI:** 10.1186/s12917-025-04521-5

**Published:** 2025-02-27

**Authors:** Alaa Emara Rabee, Afaf A. El Shereef, Mahmoud S. Nassar, Mohammed A. H. El-Rayes, Rasha S. Mohammed, Salah Abo Bakr

**Affiliations:** 1 Animal and Poultry Nutrition Department, Desert Research Center, Ministry of Agriculture and Land Reclamation, Cairo, Egypt; 2 Animal and Poultry Physiology Department, Desert Research Center, Ministry of Agriculture and Land Reclamation, Cairo, Egypt; 3 Animal and Poultry Health Department, Desert Research Center, Ministry of Agriculture and Land Reclamation, Cairo, Egypt

**Keywords:** Garlic, Barki lambs, Growth, Immunity, Rumen fermentation and microbiota

## Abstract

**Background:**

Garlic (*Allium sativum*) contains different bioactive compounds that have antimicrobial activities, which might modify rumen fermentation and improve animal performance. This study investigated the effect of garlic powder supplementation on growth performance, rumen fermentation and microbiota, blood metabolites, and immunity in Barki lambs. Twelve Barki lambs were assigned into two dietary treatments (*n* = 6): basal diet (control group) or basal diet supplemented with garlic powder as 2% of dry matter (DM) intake. The basal diet consisted of alfalfa hay and a concentrate feed mixture.

**Results:**

The results showed that garlic supplementation enhanced the growth performance, blood immunoglobulins IgG and IgA, rumen pH, and the proportions of propionic, isobutyric, and isovaleric (*P* < 0.05). Moreover, the rumen ammonia, predicted methane, and microbial alpha diversity were declined due to garlic supplementation (*P* < 0.05). Principal Co-ordinate analysis (PCoA) revealed that microbial communities were clustered according to dietary treatments. The bacterial community was dominated by phyla Bacteroidota and Firmicutes. The supplementation improved the relative abundance of the family Ruminococcaceae, genus *Prevotella* and *Butyrivibrio* which were correlated positively with growth performance and blood immunity (*P* < 0.05).

**Conclusions:**

The results explain that garlic powder could modify rumen microbiota to improve rumen fermentation, immunity, and growth performance in growing lambs.

## Background

Barki sheep is one of the main sheep breeds in the desert areas in the Mediterranean zone [1]. It provides meat and milk under poor feeding and heat stress, thus, it contributes to food security in rural desert communities [2]. However, Barki sheep did not receive enough attention compared to other ruminants. The productivity of ruminant animals, including Barki sheep in arid regions is challenged by poor nutrition and health problems [1]. Therefore, improving the performance of Barki sheep might contribute to an increase in animal products under climatic change scenarios. Improving animal performance and health using certain feed additives is considered an important solution [3]. In addition, the prohibition of antibiotics in animal feeds attracted attention towards safe and environment friendly feed additives that do not leave residues in animal products, and do not create drug resistance in pathogens. Garlic (*Allium sativum*) powder is a promising feed additive that is widely used as a source of numerous bioactive compounds such as allicin, alliin, diallyl sulfide, and diallyl trisulfide in addition to polyphenols such as hydroxybenzoic acid and p-hydroxycinnamic acid [4, 5]. Additionally, garlic is a rich source of sulfur-rich amino acids, and minerals such as sulfur, potassium, phosphorus, magnesium, sodium, and calcium.

The bioactive compounds in garlic play important roles as antioxidant, antibacterial, anti-inflammatory, and immunity stimulators [6]. Garlic has prebiotic properties also, as it has several nutrients that stimulate the gut microbial ecosystem in addition to its antimicrobial activities against bacteria, protozoa, yeast, and rumen methanogens [4]. Therefore, garlic can be used as a rumen modifier to improve rumen fermentation and animal efficiency [5]. Rumen fermentation is carried out mainly by rumen bacteria that dominate the rumen microbiota and digest a wide range of substrates such as cellulose, hemicellulose, pectin, and protein [1]. The main target of rumen fermentation modification is to increase the supply of volatile fatty acids (VFA) and microbial protein and decline methane emission, which provides more energy to the host animal [5]. Garlic powder supplementation has many fold advantages in rumen fermentation and other growth attributes. Garlic powder supplementation improved the growth performance, dry matter digestibility, total VFA, and the proportions of acetic and propionic, and declined the rumen ammonia in lambs infected by gastrointestinal nematodes [7]. Also, garlic supplementation improved blood immunity, growth rate, rumen fermentation, and digestibility of lambs [3]. Garlic powder improved rumen fermentation, blood protein and albumin with declined cholesterol concentration in ewes [8].

Garlic supplementation also altered the composition of bacterial community and declined the methane emission [5, 6, 9, 10]. Garlic skin increased the relative abundance of fiber-degrading bacteria such as *Prevotella*, *Treponema*, and *Ruminococcus* in the rumen of fistulated goats [6]. Additionally, garlic supplementation increased the relative abundance of *Prevotella*,* Bulleidia*, *Howardella*, and *Methanosphaera* and decreased the abundance of *Fretibacterium* [5]. Hence, it was aimed to investigate the effect of garlic powder supplementation on growth performance, blood metabolites, immunity, rumen fermentation, and bacteria community in the growing Barki lambs.

## Methods

### Ethics

All experimental protocols, including the euthanasia of lambs were carried out under the permission and guidelines of the Animal Ethics and Care Committee of the Division of Animal and Poultry Production, Desert Research Center, Cairo, Egypt (Project ID: AN-3-2023). All methods and protocols in this study comply with the ARRIVE 2.0 guidelines.

### Animals and diets

This study was carried out at Maryout Research Station, Desert Research Center, Alexandria, Egypt. Twelve male Barki lambs (19.33 ± 0.75 kg initial body weight, and 4–5 months of age) were selected for this 112-days experiment and divided into two groups (*n* = 6). The lambs used in this study were the offspring of the experimental sheep herd at Maryout Research Station, Desert Research Center, Alexandria, Egypt; and the animals were used in the experiment following the required consent from the administration. All animals received the same basal diet that consisted of 70% concentrates feed mixture (CFM) and 30% Alfalfa (*Medicago sativa*) that was formulated to meet the growing lambs’ feeding requirements. The hay was offered without treatments and the CFM consisted of corn 54%, soybean meal 10%, wheat bran 20.5%, cotton meal 12%, limestone 1.5%, salt 1%, Sodium bicarbonate 0.3%, Trace Minerals 0.3%, Antitoxins 0.3%, yeast 0.1%. The chemical compositions of CFM and Alfalfa hay are presented in Table 1. The control group (CC) received the basal diet with no additives, and the second group (GG) received the basal diet supplemented with garlic powder as 2% of DM intake according to the recommendation of Nassar et al. [11]. Garlic powder was mixed with concentrate feed to confirm full intake. The weights of the lambs were recorded at the beginning of the trial and biweekly to estimate growth performance as relative growth rate (RGR). Lambs were housed in shaded pens and drinking water was offered twice a day. Orts were weighed every day to determine the feed intake and diet and orts samples were dried in a forced-air oven at 60 °C for 48 h.

**Table 1:** The chemical composition of Concentrate feed mixture and Alfalfa Hay

Item	DM	OM	CP	CF	EE	NFE	ASH
Concentrate feed mixture*	85.91	93.82	14.89	6.03	3.36	69.54	6.18
Alfalfa Hay (Medicago sativa)	87.98	89.73	15.93	34.15	0.98	38.67	10.27

*Concentrate feed mixture contained of corn 54%, soybean meal 10%, wheat bran 20.5%, cotton meal 12%, lime stone 1.5%, salt 1%, Sodium bicarbonate 0.3%, Trace Minerals 0.3%, Antitoxins 0.3%, yeast 0.1%. DM = Dry matter; OM = Organic matter; CP = Crude protein; EE = Ether extract; CF = Crude fiber; NFE = Nitrogen-free extract

### Blood samples, serum metabolites and immunity

Blood samples were collected at the end of the experimental period from the jugular vein and blood serum was separated by centrifuging at 10,000× g for 5 min and the samples were frozen at -20 ^o^C for further analysis. The analysis of total protein (TP), albumin (ALB), glucose (GLU), cholesterol, (CHO), and urea (UREA) were determined using commercial kits (Spectrum Biotechnology, Egypt) according to manufacturer’s recommendations. In addition, Immunoglobulins including Immunoglobulin A, Immunoglobulin G, and Immunoglobulin M were determined using Enzyme-Linked Immunosorbent Assay (ELISA).

### Rumen fermentation parameters and predicted methane production

Rumen liquor was collected at the end of the experimental period from the lambs using an oral stomach tube. Rumen liquor was squeezed through four-layer cheesecloth; then the pH was recorded immediately using a pH meter. The rumen fluid samples were used to determine the volatile fatty acids (VFA) and ammonia nitrogen concentration (NH_3_-N) as well as DNA extraction to analyze the bacterial community. To analyze the ammonia and VFA, 1 mL of rumen fluid was centrifuged at 15,000 rpm for 20 min and the supernatant was used for VFA and ammonia measurement. Rumen ammonia was determined using an ammonia assay kit (Biodiagnostic, Cairo, Egypt) according to manufacturer recommendations. VFAs analysis was conducted using the Thermo Scientific TRACE 1300 gas chromatography system (Thermo Scientific, Massachusetts, United States), wherever nitrogen was used as the carrier gas at a flow rate of 7 ml/min, while hydrogen and make-up gases were set at flow rates of 40 ml/min and 35 ml/min, respectively. The calibration was conducted using the standards of VFAs. The predicted methane was calculated using the concentration of propionic acid, Methane yield = 316/propionate + 4.4, according to Williams et al. [12].

### Analysis of bacterial community

#### DNA extraction and PCR amplification

Microbial DNA was extracted from 0.5 ml of rumen fluid. The sample was centrifuged at 13,000 rpm and the resulting pellets were used in DNA extraction using i-genomic Stool DNA Extraction Mini Kit (iNtRON Biotechnology, Inc.) using the supplier protocol. The quality and quantity of extracted DNA were checked by gel electrophoresis and a Nanodrop spectrophotometer 2000 (Thermo Scientific, Massachusetts, United States). The bacterial community was identified using amplification of the variable V4 region on 16 S rDNA using 515 F and 926R primers through the following PCR amplification conditions: 94 °C for 3 min; 35 cycles of 94 °C for 45 s, 50 °C for 60 s, and 72 °C for 90 s; and 72 °C for 10 min. PCR products were purified and sequenced using the Illumina MiSeq system (Illumina, California, United States).

#### Bioinformatics analysis

The analyses of generated paired-end (PE) Illumina raw sequences were conducted in R (version 3.5.2) using DADA2 (version 1.11.3) [13]. The fastq files were demultiplexed and the quality checks of forward and reverse reads were conducted based on the quality scores. The clean sequence reads were denoised, dereplicated, and filtered for chimeras to generate Amplicon Sequence Variants (ASVs). Taxonomic assignment of sequence variants was conducted using a combination of the functions assign Taxonomy and assignSpecies and was compared using the latest version of SILVA reference database. The ASV table was used to calculate alpha diversity metrics, including observed ASVs, Chao1, Shannon, and Inverse Simpson. Beta diversity was determined as principal coordinate analysis (PCoA) using Bray-Curtis dissimilarity and visualized using the phyloseq and ggplot packages. The raw sequence reads are available at https://www.ncbi.nlm.nih.gov/sra/PRJNA1140745.

### Histomorphological examination of rumen samples

At the end of the experiment, the lambs in every group were sacrificed. The animals were fasted for 12 h with free access to water; and then transported to Maryout Research Station’s slaughterhouse, Maryout Research Station, Desert Research Center, Alexandria, Egypt. The animals were sacrificed by an experienced technician by cutting all the main arteries and veins in the neck with a sharp knife without electrical stimulation or using any chemical treatments. Furthermore, the animals were not anaesthetized or unconscious during the scarification. The death of the animals was ensured before the processing and sampling. Rumen autopsy samples were collected to conduct the histological examination. Rumen samples were fixed with a 10% neutral buffered formalin solution, and then washed, dehydrated in ethyl alcohol of different grades, cleared in methyl benzoate, and embedded in paraffin wax. The 5-µm sections were stained with hematoxylin and eosin and examined microscopically [14].

#### Chemical composition of diets

Animal feeds and orts samples were dried at 60 ^o^C for 48 h and were ground and analyzed according to AOAC [15] and Van Soest et al. [16] to measure DM, crude protein (CP), ether extract (EE), and crude fiber (CF).

### Statistical analysis

The differences in RGR, feed intake, blood metabolites, rumen fermentation, and the relative abundances and diversity of rumen bacteria were examined using an unpaired T-test at *P* < 0.05. Principal component analysis (PCA) and Pearson correlation analysis (Heatmap) were conducted based on the data. The statistical analyses were performed using SPSS v. 20.0 software package SPSS [17] and PAST Hammer et al. [18].

## Results

### Feed intake and growth performance

Intake of DM, OM, CP, CF, EE, and NFE (g/head/day) (Table 2) declined slightly due to garlic supplementation without significant differences (*P* > 0.05). Furthermore, growth performance expressed as RGR showed an increase in the GG group compared to the control group (*P* < 0.05).

**Table 2:** Effect of garlic supplementation on total feed intake (g/d) and growth performance (RGR)

	Control (CC)		Garlic (GG)		*P* value
	Mean	SE	Mean	SE	
DMI, g/d	949.46	31.08	918.04	35.56	0.52
OMI, g/d	878.93	28.77	849.85	32.92	0.52
CPI, g/d	143.80	4.70	139.04	5.38	0.52
CFI, g/d	138.69	4.54	134.10	5.19	0.52
EEI, g/d	25.00	0.81	24.18	0.93	0.52
NFEI, g/d	570.78	18.68	551.89	21.37	0.52
Growth performance					
IBW, kg	19.58	1.33	19.08	0.85	0.75
RGR, kg	110.91%	9.64	135.18%	4.58	0.046

DMI = Dry matter intake; OMI = Organic matter intake;

EEI = Ether extract intake; CPI = Crude protein intake;

CFI = Crude fiber intake; IBW = Initial Body weight

Relative Growth Rate (RGR), % = (final BW– initial BW) × 100/IBW

### Rumen fermentation

Rumen pH was increased significantly (*P* < 0.05) in the GG group (6.65) compared with the control group (6.37) (Table 3). Moreover, rumen ammonia was declined in group GG due to the garlic supplementation (*P* < 0.05). Total VFAs production revealed a numeric increase in group GG (*P* > 0.05). Furthermore, group GG showed higher propionate and isobutyrate (*P* < 0.05); while the control group showed higher butyrate and isovalerate (*P* < 0.05). Predicted methane was declined significantly (*P* < 0.05) due to garlic supplementation (Table 3).

**Table 3:** Effect of garlic supplementation on the rumen fermentation parameters and predicted methane

	Control (CC)		Garlic (GG)		*p* value
	Mean	SE	Mean	SE	
pH	6.37	0.08	6.65	0.05	0.032
Ammonia, mg/dL	21.34	0.73	10.20	1.21	0.0001
Acetic, mM	38.66	5.40	38.80	1.73	0.98
Propionic, mM	27.10	0.57	37.30	1.96	0.002
Isobutyric, mM	0.57	0.14	1.69	0.20	0.004
Butyric, mM	15.23	0.43	13.58	0.35	0.025
Isovaleric, mM	6.17	0.53	4.20	0.32	0.02
Valeric, mM	7.35	0.81	5.88	0.60	0.20
Total VFA, mM	95.09	6.29	101.47	3.06	0.39
Predicted methane, g /kg DMI	16.07	0.24	12.94	0.45	0.001

### Blood biochemical and immunity

The results of blood serum metabolites and immunoglobulins (Table 4) revealed similar metabolites between the groups. Moreover, garlic supplementation improved the concentration of IgA and IgG compared to the control (*P* < 0.05).

**Table 4:** Effect of garlic supplementation on the serum biochemical parameters and immunoglobulins in growing Barki lambs

	Control (CC)		Garlic (GG)		*P* value
	Mean	SE	Mean	SE	
Total Protein, g/dl	7.2	0.25	7.89	0.68	0.37
Albumin, g/dl	4.93	0.56	5.01	0.36	0.91
Globulins, g/dl	2.46	0.45	3.42	0.63	0.25
Glucose, mg/dl	91.96	4.87	89.20	4.92	0.70
Cholesterol, mg/dl	113.48	8.45	118.69	4.05	0.59
Urea, mg/dl	47.89	1.38	49.64	5.62	0.77
IgG, mg/dl	115.82	7.20	140.75	6.48	0.042
IgA, mg/dl	34.69	1.27	43.18	3.14	0.046
IgM, mg/dl	26.36	2.83	26.32	2.48	0.99

### Diversity of bacterial community

**Fig. 1:**
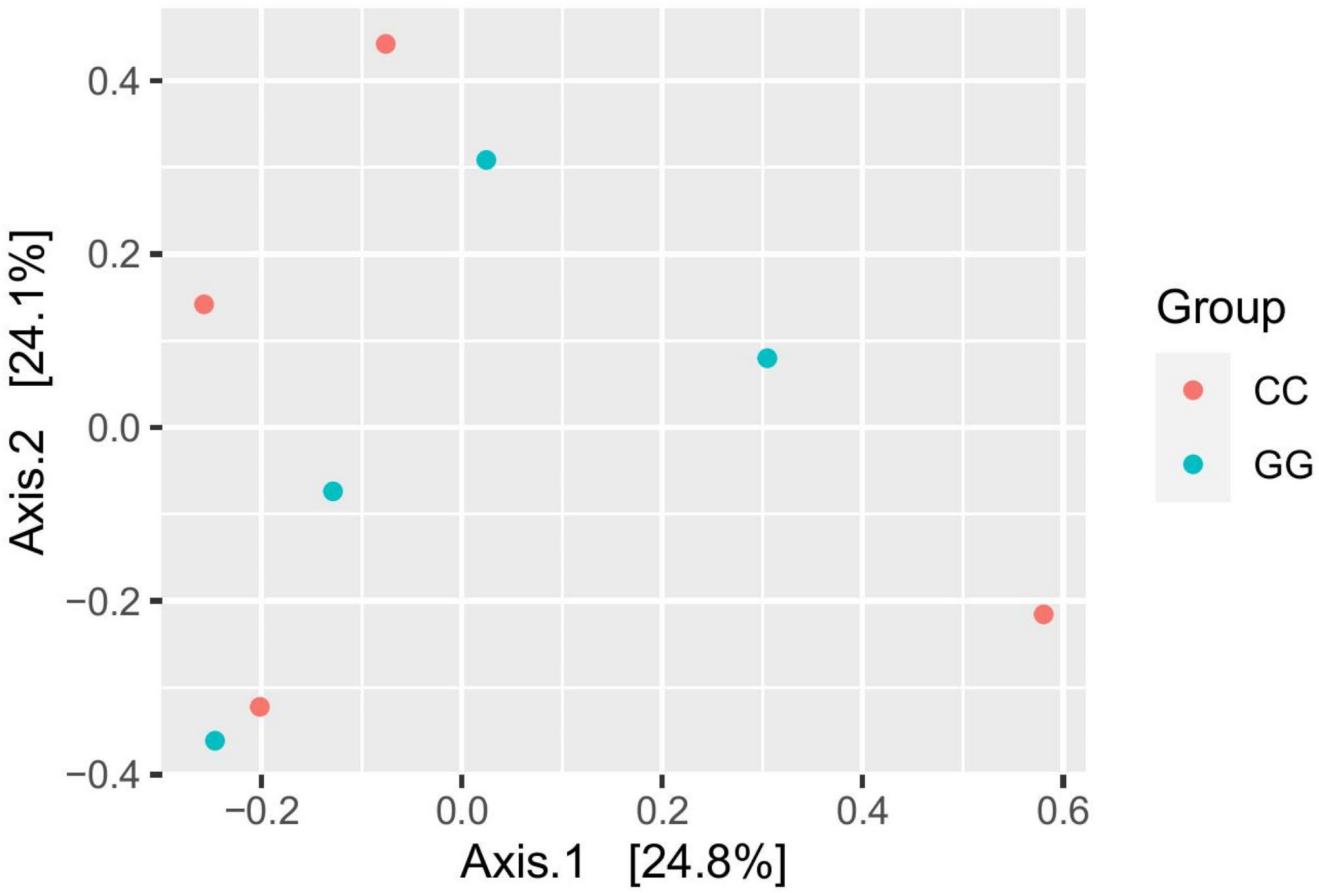
Principal coordinates analysis (PCoA). PCoA of microbial community was performed based on Bray-Curtis dissimilarity. The analyses were conducted between two lambs groups: red circles for the control group (**CC**), and blue circles for the garlic-supplemented group (GG)

The amplicon sequencing of 16 S rDNA genes generated 1,317,502 high-quality sequence reads with a mean of 164,687 reads per samples. Garlic supplementation declined the alpha diversity metrics, observed ASVs, Chao, Shannon, and Invers simpson (*P* < 0.05) (Table 5). Beta diversity of the bacterial community was measured and visualized using principal coordinate analysis (PCoA) based on Bray-Curtis dissimilarity (Fig. 1), which revealed that garlic supplementation separated rumen samples into two groups according to the treatment.

**Table 5:** Effect of garlic supplementation on the alpha diversity indices of rumen microbiota in the rumen of Barki lambs

	Control (CC)		Garlic (GG)		*P* value
	Mean	SE	Mean	SE	
Observed ASVs	675.75	84.56	423.75	36.77	0.034
Chao1	675.75	84.56	423.75	36.77	0.034
Shannon	1.42	0.16	1.98	0.08	0.021
Invers Simpson	15.57	1.60	10.11	0.25	0.015

### Composition and relative abundance of bacterial community

The Illumina sequencing failed or was weak in four samples (two per group), which were discarded from the analysis. The analysis of the bacterial community in the rumen of lambs revealed 14 bacterial phyla and one archaeal phylum (Euryarchaeota). The bacterial community was dominated by phylum Bacteroidota (72.38%), Firmicutes (24.53%), and Planctomycetota (1.12%) (Table 6). Bacterial phyla that represented less than 1% of the bacterial community were: Actinobacteriota, Chloroflexi, Cyanobacteria, Desulfobacterota, Fibrobacterota, Fusobacteriota, Proteobacteria, Spirochaetota, Synergistota, and Verrucomicrobiota. Furthermore, phylum Armatimonadota was observed only in the control group. Garlic supplementation (group GG) declined the relative abundance of Chloroflexi, Fibrobacterota, and Verrucomicrobiota (*P* < 0.05) (Table 6). The bacterial community was dominated by phylum Bacteroidota, which was dominated by families Prevotellaceae, Rikenellaceae, Muribaculaceae, F082, and Bacteroidaceae (Table 7). Family F082 was decreased by the supplementation (*P* < 0.05). Family Prevotellaceae was classified mainly to genus *Prevotella* which showed a numeric increase in group GG (*P* > 0.05). Family Rikenellaceae was dominated by genus *Rikenellaceae RC9 gut group*.

**Table 6:** Effect of garlic supplementation on the relative abundances (%) of rumen bacterial phyla

	Control (CC)		Garlic (GG)		*P* value
	Mean	SE	Mean	SE	
Actinobacteriota	0.085	0.05	0.17	0.085	0.43
Armatimonadota	0.012	0.004	00	00	00
Bacteroidota	72.78	3.32	71.99	7.93	0.93
Chloroflexi	0.14	0.041	0.04	0.009	0.048
Cyanobacteria	0.005	0.002	0.01	0.004	0.14
Desulfobacterota	0.10	0.04	0.09	0.04	0.91
Euryarchaeota	0.045	0.022	0.09	0.031	0.29
Fibrobacterota	0.009	0.003	0.002	0.00002	0.039
Firmicutes	25.05	2.865	24.01	6.63	0.89
Fusobacteriota	0.017	0.011	0.019	0.004	0.88
Planctomycetota	1.26	0.61	0.99	0.21	0.69
Proteobacteria	0.29	0.16	1.49	0.74	0.16
Spirochaetota	0.31	0.12	0.24	0.097	0.66
Synergistota	0.06	0.03	0.03	0.004	0.32
Verrucomicrobiota	0.25	0.06	0.048	0.028	0.02

**Table 7:** Effect of garlic supplementation on the relative abundances (%) of dominant bacterial families and genera

	Control (CC)		Garlic (GG)		*P* value
	Mean	SE	Mean	SE	
P: Actinobacteriota, F: Atopobiaceae					
G: Olsenella	0.067	0.054	0.10	0.057	0.67
P: Bacteroidota					
F: Prevotellaceae	60.30	6.00	62.98	9.40	0.81
G: Prevotella	58.01	6.48	61.65	9.53	0.76
G: Prevotellaceae UCG-001	1.02	0.04	0.81	0.11	0.15
F: Rikenellaceae	4.10	1.22	3.88	0.15	0.86
G: Rikenellaceae RC9 gut group	3.97	1.21	3.70	0.09	0.83
F: Muribaculaceae	3.28	1.34	5.13	1.14	0.33
F: F082	6.18	1.93	0.66	0.13	0.03
F: Bacteroidaceae	0.21	0.09	0.04	0.005	0.11
P: Desulfobacterota					
G: Desulfovibrio	0.08	0.04	0.05	0.02	0.61
G: Desulfobulbus	0.02	0.007	0.04	0.015	0.36
P: Euryarchaeota; F: Methanobacteriaceae					
G: Methanobrevibacter	0.07	0.03	0.08	0.03	0.61
P: Firmicutes					
F: Lachnospiraceae	5.80	1.27	6.25	2.59	0.88
G: Lachnospiraceae NK3A20 group	2.08	0.702	2.60	1.22	0.72
G: Shuttleworthia	0.76	0.45	0.26	0.08	0.32
G: Butyrivibrio	0.053	0.01	0.11	0.005	0.004
G: Acetitomaculum	0.22	0.07	0.24	0.026	0.78
F: Ruminococcaceae	1.42	0.16	1.98	0.079	0.02
F: Christensenellaceae	5.40	0.75	3.88	0.154	0.09
G: Christensenellaceae R-7 group	5.26	0.73	3.70	0.089	0.08
F: Selenomonadaceae	0.62	0.39	0.19	0.12	0.34
F: Oscillospiraceae	2.57	0.30	3.13	0.73	0.5
F: NK4A214 group	2.19	0.31	2.38	0.34	0.69
F: Anaerovoracaceae	1.24	0.72	1.05	0.45	0.82
P: Proteobacteria					
F: Succinivibrionaceae	0.03	0.006	0.08	0.04	0.24
G: Succinivibrionaceae UCG-001	0.02	0.009	0.08	0.043	0.23
P: Spirochaetota					
G: Sphaerochaeta	0.03	0.010	0.04	0.01	0.66
G: Treponema	0.21	0.09	0.14	0.04	0.54

*P* = phylum; F = family; G = genus

The members of phylum Firmicutes were classified mainly into families Lachnospiraceae, Ruminococcaceae, Christensenellaceae, Selenomonadaceae, Oscillospiraceae, NK4A214 group, and Anaerovoracaceae. Family Lachnospiraceae was affiliated to *Lachnospiraceae NK3A20 group*, *Shuttleworthia*, *Butyrivibrio*, and *Acetitomaculum*. Genus *Butyrivibrio* revealed higher relative abundance in group GG (*P* < 0.05) (Table 7). The relative abundance of family Ruminococcaceae was improved due to garlic supplementation (group GG) (*P* < 0.05). Family Christensenellaceae was assigned to the genus *Christensenellaceae R-7 group*. Phylum Proteobacteria was affiliated mainly to the family Succinivibrionaceae and genus *Succinivibrionaceae UCG-001*. Phylum Spirochaetota was dominated by the genus *Sphaerochaeta* and *Treponema* (Table 7). Phylum Euryarchaeota was classified into the genus *Methanobrevibacter*.

### Principal component analysis (PCA)

**Fig. 2:**
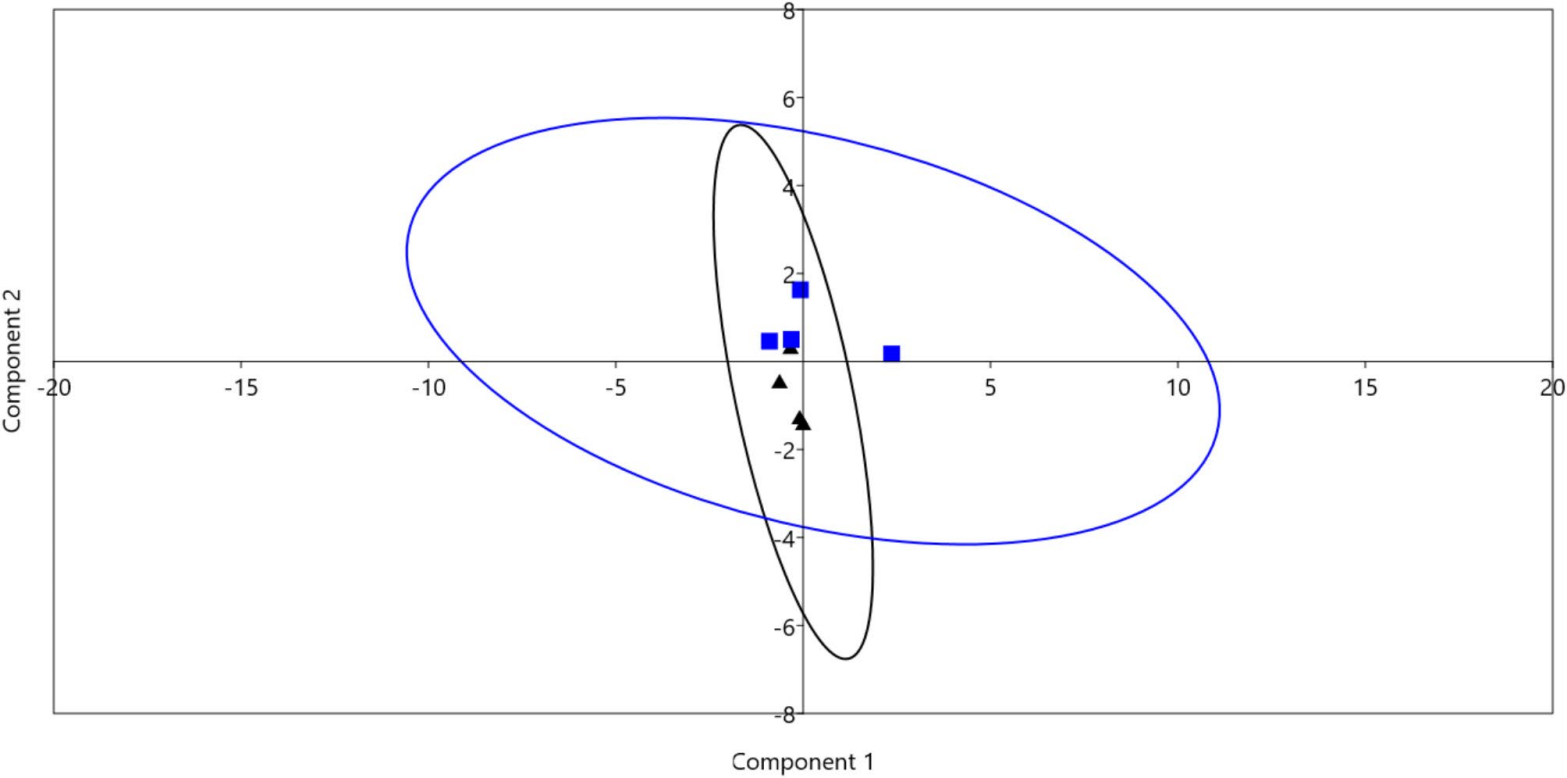
Principal component analysis (PCA). The PCA analysis was determined using RGR, rumen fermentation parameters, and the relative abundances of dominant bacterial phyla, families, and genera. The blue squares for control group (CC) and black triangles for garlic-supplemented groups

PCA analysis was performed using the data of RGR, rumen fermentation parameters, blood metabolites, and the relative abundances of dominant bacterial phyla, families, and genera (Fig. 2). The results revealed that the samples were separated based on the treatment. The separation was driven by total VFA, propionic acid, blood metabolites, and the relative abundances of phyla Bacteroidota, Firmicutes, and genus *Prevotella*.

### Pearson correlation analysis

**Fig. 3:**
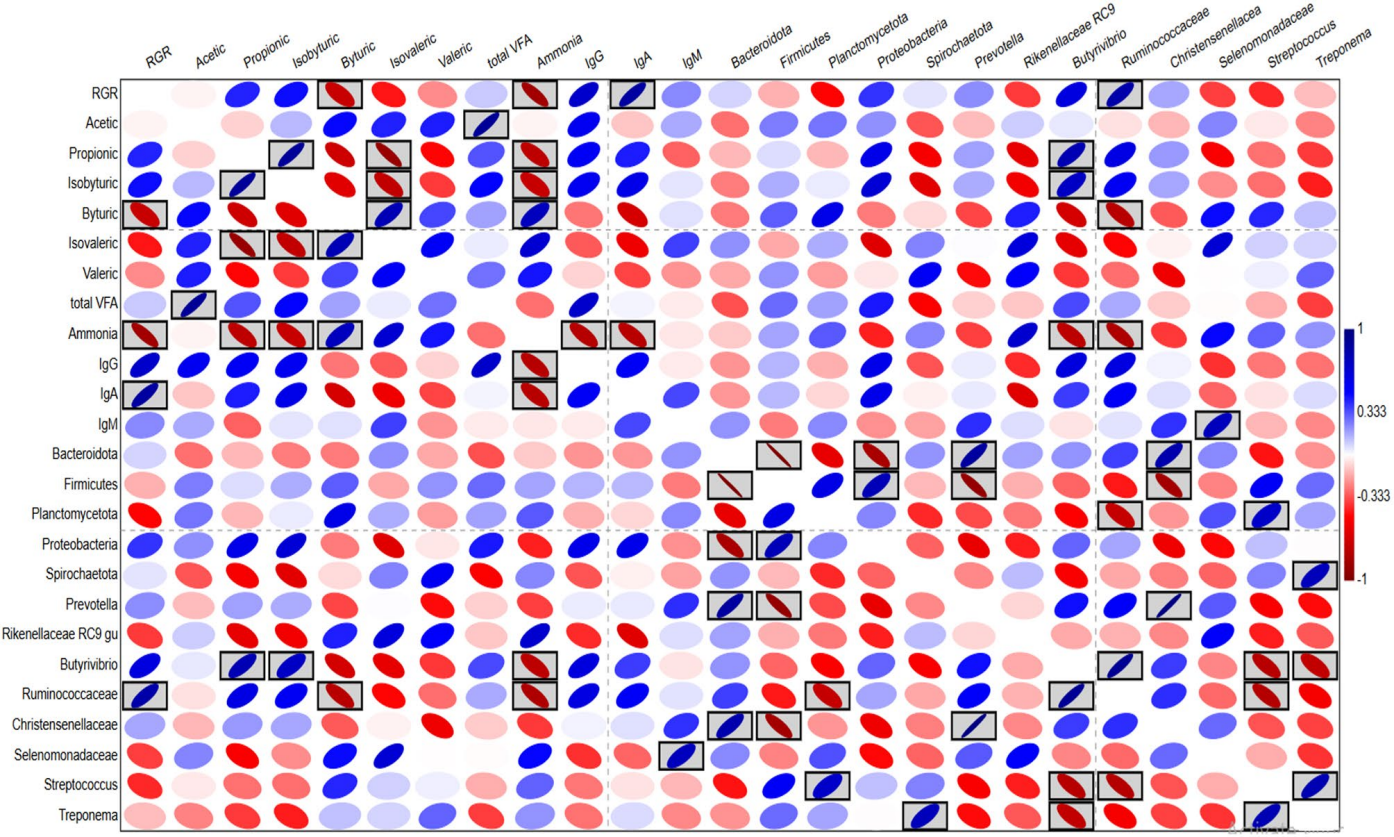
Pearson correlation analysis visualized as a heatmap. The correlation relationships were conducted between RGR, rumen fermentation parameters, and the relative abundances of dominant bacterial phyla, families, and genera

Pearson correlation was determined using the data of RGR, rumen fermentation parameters, blood metabolites, immunity, and the relative abundances of dominant bacterial groups (Fig. 3). The correlation relationships were visualized in the heatmap (Fig. 3). The analysis revealed some positive and negative correlation relationships. For example, growth performance (RGR) was correlated positively with propionic, isobutyric, IgG, IgA, Proteobacteria, *Prevotella*, *Butyrivibrio*, and Ruminococcaceae. Furthermore, there was a positive correlation between the genus *Prevotella* and RGR and propionic acid. Genus *Butyrivibrio* was correlated positively with TVFA production.

#### Histology of the rumen tissues

**Fig. 4:**
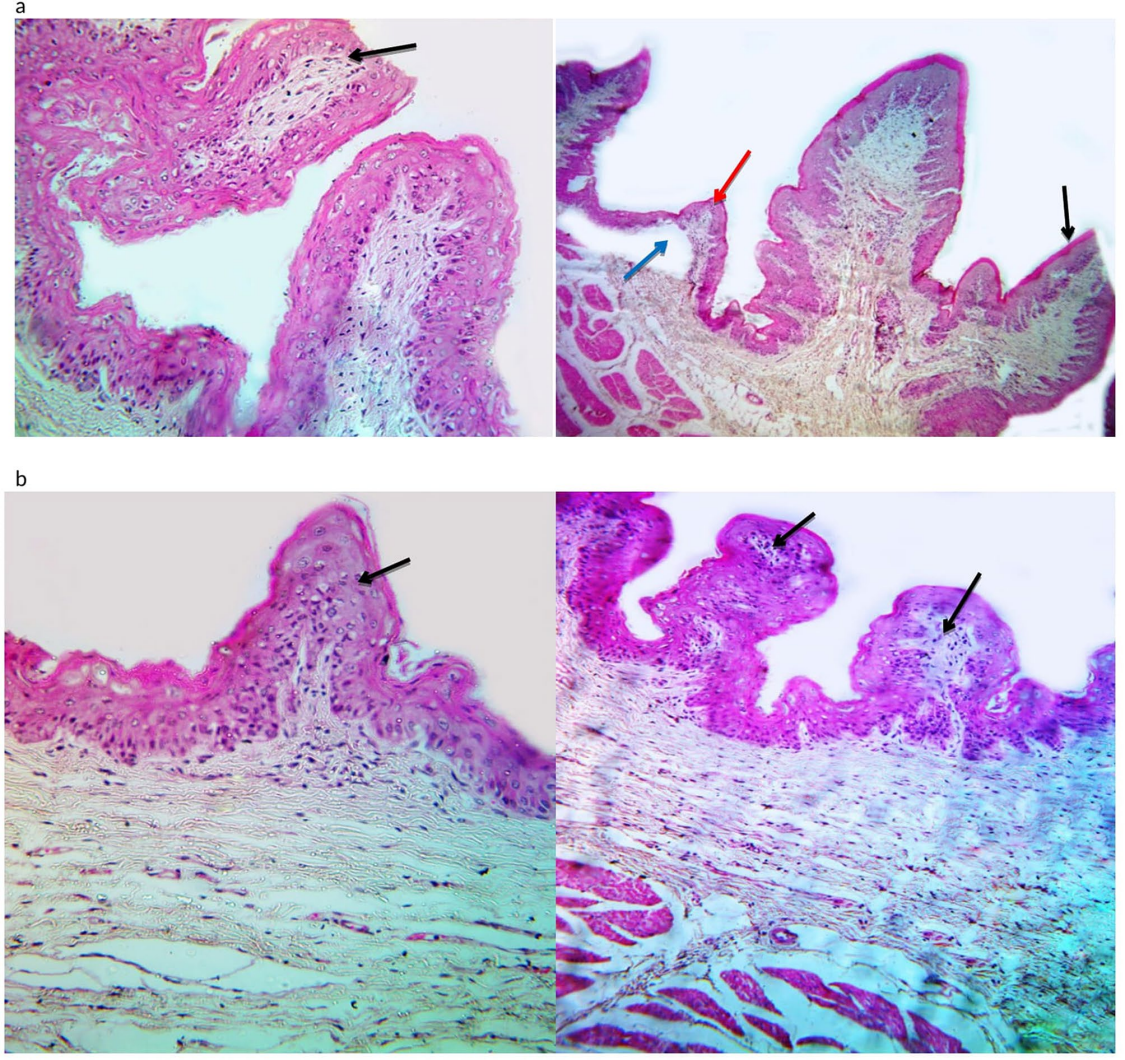
The histology of rumen papillae in Barki lambs supplemented by garlic powder. **a:** refers to the control group and shows degeneration and atrophy of the apex of ruminal papillae (black arrow), shortness of ruminal papillae (red arrow), and clear edema (blue arrow). **b:** refers to papillae of garlic-supplemented lambs and showing shortness of ruminal papillae, and slight infiltration of inflammatory cells (black arrow)

The histology of rumen papillae varied in the garlic-supplemented group (GG) (Fig. 4). A slight degeneration and atrophy of the apex of ruminal papillae were detected in the rumen papillae of the control group (Fig. 4).

## Discussion

### Animal performance and rumen fermentation

The herbal plants’ supplementation is an emerging strategy to improve animal performance and a safe alternative to synthetic drugs [3]. Garlic supplementation modified the rumen ecosystem and enhanced blood immunity, which led to improved animal performance. Feed intake was similar between animal groups, which agrees with previous studies [3, 5, 7]. The increment in the growth performance due to garlic supplementation (Table 2) was also indicated by previous studies on sheep [3, 7, 11]. On the other side, Chaves et al. [19] noted no changes in the growth due to garlic supplementation. Zhu et al. [5] explained that garlic supplementation improves the synthesis of vitamin B6, which improves the digestibility in animals. Furthermore, previous studies [5, 20] reported that garlic supplementation improved the synthesis of microbial protein and VFA, which enhanced the protein and energy supply to the host animal. In the same line, Kewan et al. [3] indicated that garlic supplementation enhanced the digestibility of nutrients except for the digestibility of crude protein. Similar studies on growing goats and sheep [7, 21] explained that garlic supplementation declined the parasites count in infected lambs, which improved the growth performance. Chitwood [22] demonstrated that sulfur compounds in garlic control the parasites by suppressing their physiological processes. On the other hand, previous studies [3, 7, 21] reported that garlic powder stimulates animal’s immunity to resist parasites and pathogens. These findings are consistent with the increment in blood immunoglobulins in the current study (Table 4). Therefore the improvement in growth performance in the garlic-supplemented group could be attributed to improved feed utilization and immunity. This finding was confirmed by the positive correlation between growth performance and blood immunoglobulins (Fig. 3).

Previous studies [5, 20] reported changes in the rumen fermentations due to garlic supplementation, which supports the current study (Table 5). Rumen pH was improved (from 6.37 (CC) to 6.65 (GG)) due to garlic supplementation, which agrees with previous studies used garlic straw or garlic extract [10, 23]. Zhu et al. [5] noted no changes in rumen pH when supplemented lambs with garlic skin. Increased rumen pH stimulates the activities of fiber-degrading bacteria that require neutral pH (> 6.5) [24]. Rumen ammonia was declined due to the supplementation, which was indicated by previous studies [3, 5, 7, 8]. The decline in the ammonia indicates an increased incorporation of ammonia in microbial protein or lower ruminal protein degradation, which increases the bypass protein to the intestine, which improve the animal performance [25]. This speculation was confirmed by earlier reports [5, 20] that reported improved microbial population and synthesis of microbial protein due to garlic supplementation. Furthermore, the increment in TVFA and propionic acid was also reported by previous studies [5, 7, 10, 26]. The increment in VFAs indicates that garlic has a positive effect on rumen fermentation, which increases the energy supply to the host animal and improves its performance [27]. The decline in methane production due to garlic supplementation was also indicated in previous studies on garlic [3, 20, 28]. Kewan et al. [3] reported that garlic supplementation declined the counts of rumen protozoa. The majority of rumen methanogens are closely associated with protozoa that provide hydrogen and growth substrates to rumen methanogens [4]. Ma et al. [29] reported that allicin (an active ingredient in garlic) declined the methanogens population and methane production, which supports the current findings. Methane represents a loss in gross energy feed intake of host animals as well as a source of global warming, therefore, declining methane could increase the animal’s feed efficiency and cut emissions from the livestock sector [4, 27]. These explanations could justify the improvements in the growth performance of lambs in the current study.

### Bacterial community

Rumen microbiota work together to ferment ingested feed to produce VFAs that represent the major source of energy for the host animals. Therefore, any modulations in the rumen microbiome affect the fermentation of animal diet [27]. In this study, the improvements in rumen fermentation, blood immunity, and growth performance were associated with variations in the rumen microbial community due to the supplementation. This finding was confirmed by the results of PCoA, PCA, and Pearson correlation analyses (Fig. 1). The supplementation declined the microbial alpha diversity and impacted the samples’ clustering in Beta diversity (PCoA) (Table 5; Fig. 1). A similar finding was observed in earlier studies [5, 6], where the decline in microbial diversity was attributed to the antimicrobial properties of garlic that modify the rumen ecosystem. The declined microbial alpha diversity was associated with higher feed efficiency [30]. Bacteriodetes and Firmicutes dominated the microbial community (Table 6), which agrees with the previous findings on garlic [5, 6] on lambs and goats fed garlic skin. Moreover, the bacterial community was dominated by genus *Prevotella* (Table 7), which includes members active in the fermentation of different substrates such as hemicellulose and protein [31]. Furthermore, *Prevotella* produces propionate that consumes the hydrogen molecules, which reduces its availability for methane production by methanogenic archaea [32]. These findings explain the positive correlation between *Prevotella* and RGR and propionic acid. Zhu et al. [5] indicated that *Prevotella* was increased by the garlic supplementation, which supports the previous explanation.

Garlic supplementation improved the relative abundance of family Ruminococcaceae and genus *Butyrivibrio* (Phylum Firmicutes). This finding was also reported by Gu et al. [6] who reported an increment in the genus *Ruminococcus* (a member of family Ruminococcaceae). Family Ruminococcaceae and genus *Butyrivibrio* play an important role in fiber and protein digestion which improves the VFA supply and animal feed efficiency [33, 34]. This finding was supported by the positive correlation between *Butyrivibrio* and TVFA production. Furthermore, cellulolytic bacteria such as *Butyrivibrio* and Ruminococcaceae represent a barrier against pathogens, which boosts animal immunity [35]. These findings were supported by the positive correlation between RGR, blood immunoglobulins, and Family Ruminococcaceae and genus *Butyrivibrio* (Fig. 3).

### Histology of the rumen papillae

The garlic supplementation improved the morphology of rumen papillae in the garlic-supplemented lambs, which agrees with sheep supplemented by garlic leaf [36] and goats supplemented by herbal mixture, including garlic powder [27]. Redoy et al. [36] indicated that the improvements in the morphology of rumen papillae could be attributed to higher production of propionic and butyric acids, which supports the current finding. Petric et al. [37] indicated that the absorption of VFA through rumen epithelium is affected by the surface area of the ruminal papillae, which highlights the positive effect of garlic supplementation on the rumen papillae.

## Conclusion

Garlic powder could be used supplemented at 2% of DM intake to improve the performance and immune status of growing lambs. It modifies rumen ecosystem by increasing count of fiber-degrading bacteria, which improves rumen metabolism and VFAs production.

## Data Availability

The datasets generated and/or analyzed during the current study are available in SRA at https://www.ncbi.nlm.nih.gov/sra/PRJNA1140745.

## Abbreviations

ALB: Albumin
ASVs: Amplicon Sequence Variants
CHO: Cholesterol
CF: Crude fiber
CP: Crude protein
DM: Dry matter
EE: Ether extract
GLU: Glucose
PCoA: Principal Co-ordinate analysis
PCA: Principal component analysis
RGR: Relative growth rate
TP: Total protein
VFA: Volatile fatty acids
